# Cardiogenic shock following blunt chest trauma

**DOI:** 10.4103/0974-2700.70772

**Published:** 2010

**Authors:** Fayna Rodríguez-González, Efrén Martínez-Quintana

**Affiliations:** Intensive Medicine Service, Cardiology Service, Complejo Hospitalario Universitario Insular-Materno Infantil, Las Palmas de Gran Canaria, Spain

**Keywords:** Aneurysm, blunt cardiac injury, cardiac contusion, cardiac complications, echocardiography

## Abstract

Cardiac contusion, usually caused by blunt chest trauma, has been recognized with increased frequency over the past decades. Traffic accidents are the most frequent cause of cardiac contusions resulting from a direct blow to the chest. Other causes of blunt cardiac injury are numerous and include violent fall impacts, interpersonal aggression, explosions, and various types of high-risk sports. Myocardial contusion is difficult to diagnose; clinical presentation varies greatly, ranging from lack of symptoms to cardiogenic shock and arrhythmia. Although death is rare, cardiac contusion can be fatal. We present a case of cardiac contusion due to blunt chest trauma secondary to a fall impact, which manifested as cardiogenic shock.

## INTRODUCTION

The incidence of cardiac contusion in patients with blunt chest trauma is not well known. Depending on the criteria used for diagnosis, the reported incidence ranges from 5 to 50% of blunt chest trauma cases.[1] Injury to the various structures of the heart occurs as a result of different mechanisms such as direct injury, sudden deceleration, or compression between thoracic structures. Presentation ranges from no cardiac symptoms to catastrophic injuries affecting any or all areas of the heart.[2] Myocardial contusion is the most common form of blunt cardiac injury.[3]

## CASE REPORT

**Figure 1:**
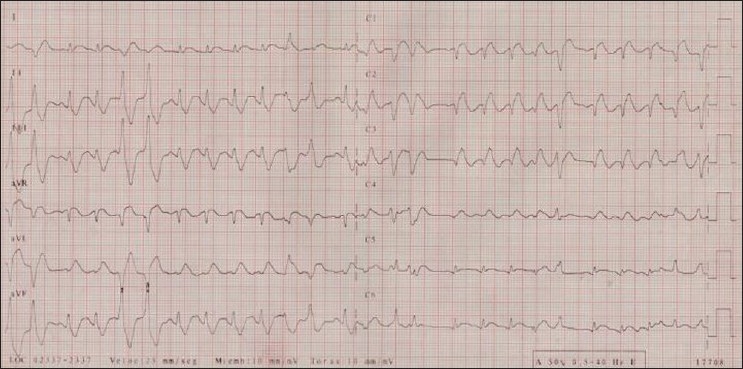
Electrocardiogram on admission depicting 1 mm ST segment elevation in lateral leads with associated right bundle branch block and ventricular beats in couplet

A 47-year-old man was referred to our hospital after a fall impact from a height of 6 m. On admission, the patient was awake and reported abdominal and thoracic pain. During clinical evaluation, he presented a cardiac arrest due to ventricular fibrillation that required four cardiac defibrillations, intubation, and mechanical ventilation. The patient recovered sinus rhythm but again presented hemodynamic instability with bilateral lung hypoventilation at auscultation. Tension pneumothorax was suspected and thoracic tubes were inserted into both sides of the chest. Episodes of ventricular tachycardia were treated with amiodarone and a complete atrioventricular block with a transitory pacemaker. A radiograph of the chest showed inferior left and right costal fractures, and free pelvic and abdominal liquid was observed by abdominal ultrasound. An exploratory laparotomy was conducted, proceeding to splenectomy due to spleen rupture. The electrocardiogram showed a 1-mm ST segment elevation in lateral leads with associated right bundle branch block [Figure 1].

The patient was admitted to the Intensive Care Unit. After 24 hours, he presented sudden hemodynamic deterioration with associated ventricular tachycardia. Pneumothorax recurrence was ruled out. Echocardiography demonstrated severe left ventricular dysfunction (ejection fraction 30%) with anteroseptal akinesia, and apical aneurysm without valve regurgitation, aortic dissection, or pericardial effusion. Upon insertion of a Swan-Ganz catheter, it was determined that the patient had a low cardiac index, elevated systemic vascular resistance, and high wedge capillary pressure. Serum creatine-phosphokinase reached 7233 IU/l with an MB fraction of 284 IU/l.

The patient’s progress was slow, but satisfactory. Catecholamines were withdrawn after 15 days, although an echocardiogram showed no improvement in ventricular function. The patient was discharged to a hospitalization room on the 43^rd^ day after admission, and sent home 15 days later.

**Figure 2:**
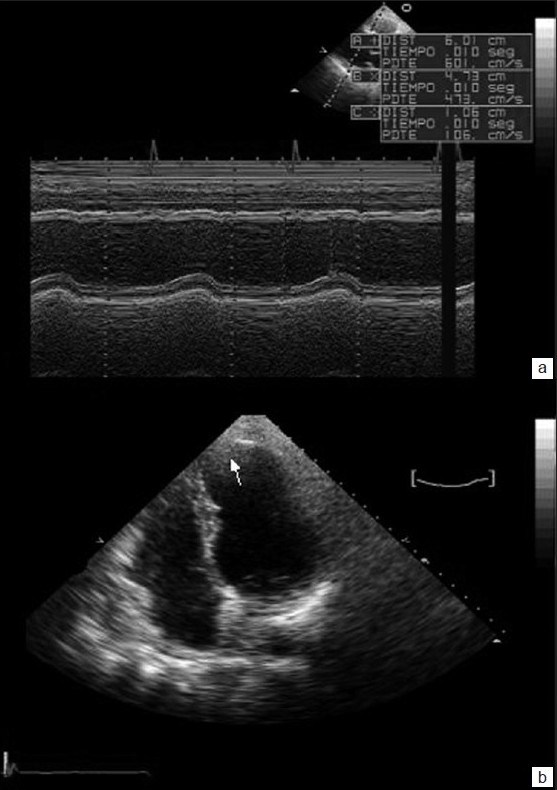
(a) M-mode obtained from the left paraesternal long axis view reveals no interventricular septum thickness and normal contraction of the posterior wall; (b) apical four-chamber 2-D echocardiogram with apical aneurysm (arrow)

Four years after the accident, the patient is in New York Heart Association functional class II/IV and is undergoing treatment with beta blockers and angiotensin-converting enzyme inhibitors. Echocardiogram shows moderate left ventricular dysfunction (ejection fraction 37%) with persistent anteroseptal akinesia and apical aneurysm [Figure 2]. The treadmill test is clinically and electrocardiographically negative.

## DISCUSSION

Traffic accidents are the most frequent cause of blunt cardiac injury, followed by violent fall impacts, interpersonal aggression, and various kinds of high-risk sports.[4] A direct blow to the chest, in combination with the direct transfer of energy during impact, can cause a sudden, forceful deceleration and compression of the heart between the sternum and the spine. The clinical presentation of a cardiac contusion varies greatly, ranging from lack of symptoms to life-threatening arrhythmias and heart failure. Cardiogenic shock and death are rarely encountered manifestations.[5]

Electrocardiographic abnormalities frequently occur in cases of myocardial contusion. However, a normal electrocardiogram alone does not exclude the diagnosis. Left ventricular injury can produce ST segment changes, as well as T wave or Q wave abnormalities. Damage to the right ventricle may cause right bundle branch block, although such a block is usually transient. Different degrees of atrioventricular block have also been described, although they are less common.[5] Arrhythmias tend to occur as a result of abnormal perfusion patterns, while conduction anomalies may happen due to damaged myocytes or vagal-sympathetic reflexes.[6] In our patient, the existence of an ST segment elevation in lateral leads, a right bundle branch block, and a complete atrioventricular block may indicate a larger and more severe myocardial contusion with a higher risk of myocardial necrosis, aneurism formation, rupture, and fatal atrial or ventricular arrhythmia.

The level of creatine kinase (CK) is nonspecifically increased in cases of cardiac trauma. Although the CK-myoglobin fraction is known to have better specificity in polytraumatized patients, many false positives have been reported. Serum cardiac troponins are highly specific to myocardial injury; however, we did not obtain troponin levels in this case because they were unavailable at the time of admission.

Echocardiography provides a view of ventricular function and diameter, associated valvular lesions, intracardiac shunts or thrombosis, and pericardial effusion or tamponade. Valvular lesions occur in less than 1% of blunt chest trauma cases and predominantly affect the aortic and mitral valves.[7] Autopsy series have revealed lesions of the coronary arteries (e.g., laceration, thrombosis, or dissection) in less than 2% of cases.[7] However, typical ischemic lesions that present as subendocardial-to-transmural damage involving the distribution territory of a coronary artery, especially when combined with elevated troponin concentration and echocardiographic data of ventricular dysfunction, are a clear indicator of myocardial infarction and should lead to emergency coronary angiography. In our case, angiography was not conducted due to the patient’s hemorrhagic shock and the lack of a cardiac catheterization laboratory in our hospital at the time.

Severe cardiac injury with myocardial necrosis heals by scar formation, similar to the cardiac scarring observed in myocardial infarction. As in our patient, cardiac scarring may lead to ventricular aneurysm formation,[8] and in some cases to chronic heart failure, and cardiac arrhythmias. These patients should be carefully evaluated to establish the necessary treatment and to avoid long-term complications.[9,10] Early diagnosis by continuous electrocardiographic monitoring, serial electrocardiograms, serum determination of cardiac markers, echocardiography, and angiography will enable the clinician to make a better assessment of the patient, thereby improving survival.

## CONCLUSIONS

In cases of blunt chest trauma, the diagnosis of a cardiac contusion must be suspected. Electrocardiographic abnormalities, increase in serum cardiac enzymes, and echocardiographic ventricular dysfunction identify patients who are at significant risk of developing cardiogenic shock, ventricular arrhythmias, and aneurism. Patients should be closely monitored in the early stages to treat any complication, and during the follow-up period to avoid sudden cardiac death and reduce symptoms of heart failure.
